# Measuring the Quality of Mobile Apps for the Management of Pain: Systematic Search and Evaluation Using the Mobile App Rating Scale

**DOI:** 10.2196/10718

**Published:** 2018-10-25

**Authors:** Alejandro Salazar, Helena de Sola, Inmaculada Failde, Jose Antonio Moral-Munoz

**Affiliations:** 1 Institute of Research and Innovation in Biomedical Sciences of the Province of Cadiz Cádiz Spain; 2 The Observatory of Pain University of Cádiz Cádiz Spain; 3 Department of Statistics and Operational Research University of Cádiz Cádiz Spain; 4 Preventive Medicine and Public Health Area Cádiz Spain; 5 Department of Nursing and Physiotherapy University of Cádiz Spain

**Keywords:** mobile app, chronic pain, Mobile App Rating Scale, mHealth, mobile phones

## Abstract

**Background:**

Chronic pain is a major health issue requiring an approach that not only considers medication, but also many other factors included in the biopsychosocial model of pain. New technologies, such as mobile apps, are tools to address these factors, although in many cases they lack proven quality or are not based on scientific evidence, so it is necessary to review and measure their quality.

**Objective:**

The aim is to evaluate and measure the quality of mobile apps for the management of pain using the Mobile App Rating Scale (MARS).

**Methods:**

This study included 18 pain-related mobile apps from the App Store and Play Store. The MARS was administered to measure their quality. We list the scores (of each section and the final score) of every app and we report the mean score (and standard deviation) for an overall vision of the quality of the pain-related apps. We compare the section scores between the groups defined according to the tertiles via analysis of variance (ANOVA) or Kruskal-Wallis test, depending on the normality of the distribution (Shapiro-Wilk test).

**Results:**

The global quality ranged from 1.74 (worst app) to 4.35 (best app). Overall, the 18 apps obtained a mean score of 3.17 (SD 0.75). The best-rated sections were functionality (mean 3.92, SD 0.72), esthetics (mean 3.29, SD 1.05), and engagement (mean 2.87, SD 1.14), whereas the worst rated were app specific (mean 2.48, SD 1.00), information (mean 2.52, SD 0.82), and app subjective quality (mean 2.68, SD 1.22). The main differences between tertiles were found on app subjective quality, engagement, esthetics, and app specific.

**Conclusions:**

Current pain-related apps are of a certain quality mainly regarding their technical aspects, although they fail to offer information and have an impact on the user. Most apps are not based on scientific evidence, have not been rigorously tested, and the confidentiality of the information collected is not guaranteed. Future apps would need to improve these aspects and exploit the capabilities of current devices.

## Introduction

Pain is defined by the International Association for the Study of Pain (IASP) as a “distressing experience associated with actual or potential tissue damage, with sensory, emotional, cognitive, and social components” [1]. This problem may eventually become chronic, in which case it should be seen as a disease rather than as a symptom. In this case, it is called “chronic pain,” defined by the IASP as pain that has persisted beyond the normal tissue healing time. The IASP proposes 3 months as a convenient cut-off point [2]. Chronic pain is a major public health problem that must be addressed, with prevalence ranging from 10% to 40% in different countries and populations [3-8].

**Figure 1:**
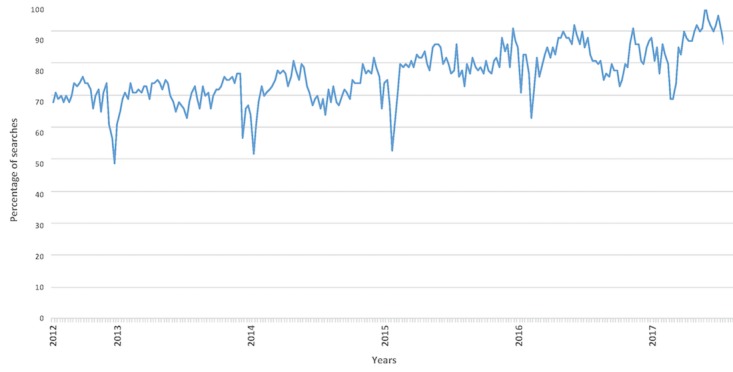
Interest over time in the term “chronic pain.” Results obtained through Google Trends. The values, expressed in percentages, reflect the number of searches done for the term relative to the total number of searches done on Google over time.

“Chronic” and “pain” have been constantly popular keywords on search engines over the last 5 years, which could be related to the increasing prevalence of this condition in recent years [9,10]. In relation to this issue, Figure 1 shows the trend of searches for “chronic pain” on Google. It is noteworthy that the largest volume of searches occurred throughout 2017, indicating that it is currently a major concern.

Treating chronic pain is a tricky task because it should involve much more than simply drugs. The biopsychosocial model states that pain is “a dynamic interaction among and within the biological, psychological, and social factors unique to each individual” [11]. In fact, chronic pain is related to many other factors, such as sick leave or job loss [12], cognitive impairment [13], perception of a negative impact on the family and social environment [3], anxiety, depression [3], and stress [14], and it also has work, social, and family-related consequences [5,12,15]. All these factors should be taken into account when treating chronic pain. In view of this, there is a need for new ways of dealing with chronic pain that can include these factors, and new technologies to support the assessment and tracking of chronic pain, as they include the social sphere, can assess and modify daily activities, and can focus on factors such as mood status.

The number of mobile health apps has grown recently, having been classified as an “exploding market,” with more than 100,000 specific apps [16]. The possibilities offered by new devices and their sensors can be very useful for this type of app [17], although they are not being well exploited.

Mobile apps specifically for pain have also increased, with there now being over 350 apps according to the review by Portelli and Eldred [18], which is an extension of a previous review [19]. However, in these articles the authors concluded that one of the main problems with the existing apps is the lack of scientific value, effectiveness testing, and evidence-based results and conclusions. The biopsychosocial approach is not included in most of the apps because social support, for instance, is not usually implemented by the software developers. Nevertheless, there are some limitations of these reviews, mainly related to the use of a checklist, which is not a validated tool to measure the quality of mobile phone apps. Therefore, there is a need for an adequate tool to measure the quality of the apps, although it does not seem that the existing apps are of a high quality or meet all the expected requirements. In some cases, it might be even harmful to trust them [20,21].

The traditional systems to try to measure the quality of the apps include the opinions and/or satisfaction of the users, the stars rating system, the app description, or checklists. None of these strategies seems to be adequate to scientifically measure the quality of the apps. For instance, the descriptions of the apps in the stores may be incomplete or imprecise, the scores and opinions may include the subjectivity of nonexpert users or be based on the opinion of very few people making it difficult to generalize, and checklists do not actually assess quality [15].

There is an alternative tool for assessing the quality of health mobile apps, namely the Mobile App Rating Scale (MARS) [17], which, to the best of our knowledge, has not been used previously to measure the quality of pain-related apps in any review. The psychometric properties of this scale have been proven, and it has been shown to be a simple, objective, and reliable tool to measure the quality of apps [17] (see the Methods section for a more detailed explanation of the scale). This is the reason for using this tool in this evaluation, and we believe that it provides greater strength to the results obtained compared to previous studies.

Finally, given the changing nature of the apps market, there is always a need for an updated review which includes the new apps that may have been released recently. In view of the preceding, this study aims to evaluate and measure the quality of mobile apps for the management of pain using the MARS.

## Methods

This study included pain-related mobile apps (both free and paid) found in the official stores of Apple iPhone (App Store) and Android (Play Store) in June 2017. These two systems are the most widely used according to the latest report by Kitagawa et al [22], accounting for 99.6% of all mobile phone sales in the fourth quarter of 2016. The search was carried out in both English and Spanish.

Firstly, we defined the disease of interest using the following generic terms: “pain” and “*dolor* ” (Spanish for “pain”). Then, the results were refined using specific terms such as “chronic pain” and “*dolor crónico*.” The apps focused on specific pain conditions; those that were not available or presented major technical errors were excluded.

A total of 18 apps were finally included (2 from the App Store, 11 from the Play Store, and 5 multiplatform). These apps were randomly divided into three groups, each of which was assigned to two reviewers, who downloaded the assigned apps on their devices, used, and evaluated them by means of the MARS [17]. A total of three reviewers were involved, so that each of them could review two groups of apps to avoid the potential subjectivity of a single reviewer. The online platform SurveyMonkey was used to help them complete the MARS.

The MARS [17] consists of 23 items grouped into the following sections: engagement, functionality, esthetics, information quality, and subjective quality. There are also six final items (app specific) that can be adapted to include or exclude specific information on the topic of interest, as well as an initial section collecting general and technical information on the app. Each item is scored from 1 (inadequate) to 5 (excellent), and a final mean score is given for each section. Finally, the mean values of the first four sections (ie, engagement, functionality, esthetics, and information quality) are used to give a final measurement of the app quality, which is the average value of the four means. The complete structure of the scale can be seen in Table 1.

The discrepancies in the scores between reviewers were assessed and if major differences were found in a specific app (more than 2 points of difference), the items of the MARS were compared. In case of disagreement, the third reviewer intervened to evaluate and reach a consensus, except for one app (Change Pain), which was no longer available when the third reviewer tried to evaluate it and was eventually removed from the study. The final score for each app was calculated as the mean of the scores of each reviewer, after verifying that the scores were similar and that there was consensus. The apps were then classified as worst-rated apps, average apps, and best-rated apps according to the tertiles of the final scores. This classification based on tertiles gave us a cut-off point for an app to be considered a best-rated app.

A descriptive analysis was performed. We list the scores (of each section and the final score) of every app and we report the mean score (and standard deviation) for an overall vision of the quality of the pain-related apps. Additionally, we compare the section scores between the groups defined according to the tertiles via analysis of variance (ANOVA) or Kruskal-Wallis tests, depending on the normality of the distribution (Shapiro-Wilk test). The analyses were performed with SPSS version 21, and the figures with Excel 2016.

**Table 1:** Structure of the MARS.

Section	Definition
A: Engagement	Fun, interesting, customizable, interactive (eg, sends alerts, messages, reminders, feedback, enables sharing), well-targeted to audience
B: Functionality	App functioning, easy to learn, navigation, flow logic, and gestural design of app
C: Esthetics	Graphic design, overall visual appeal, color scheme, and stylistic consistency
D: Information	Contains high-quality information (eg, text, feedback, measures, and references) from a credible source. Select N/A if the app component is irrelevant
App quality	Mean score of sections A, B, C, and D
E: App subjective quality	Personal interest in the app
F: App specific	Perceived impact of the app on the knowledge, attitudes, and intentions to change of the users, as well as the likelihood of actual change in the target health behavior

## Results

**Figure 2:**
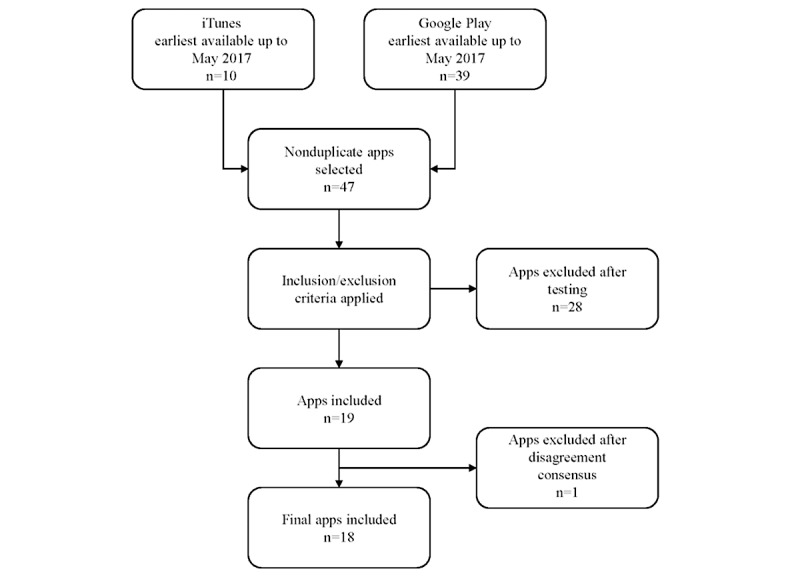
Flowchart of the pain-related app selection.

A total of 47 nonduplicate apps were initially identified as potential pain apps to be included in this study. Of these, 28 did not meet the inclusion criteria and were excluded, making a total of 19 apps analyzed, although one of the apps was eventually removed due it being unavailable in the stores when trying to solve the lack of consensus. Therefore, a final total of 18 apps were included (Figure 2; Table 2).

Table 3 shows the main characteristics of the apps included in the study. According to the users, on a scale of 1 to 5 stars, the apps had a quality score ranging from 1 to 4.8, with a mean score of 3.61 (SD 0.93). Generally, the paid apps received higher ratings, usually between 4 and 5 stars, although some free apps were also highly rated. The affiliation of most of the apps was commercial, but three of them came from university environments. The apps were mainly focused on physical health, and sometimes on depression/anxiety, increasing happiness/well-being, or reducing negative emotions. The most frequent theoretical background or strategies of the apps were monitoring/tracking, assessment, feedback, and information/education. Finally, their technical aspects included sharing options (eg, Facebook, Twitter), app communities, reminders, password protection, and log-in or Web connection required.

The specific scores for each app are shown in Table 4. The mean app quality score ranged from 1.74 (worst-rated app) to 4.35 (best-rated app), and a similar situation was observed in each section: 1.20 to 4.60 (engagement), 1.88 to 4.75 (functionality), 1.83 to 4.83 (esthetics), 1.14 to 4.00 (information), 1.00 to 4.38 (app subjective quality), and 1.00 to 4.42 (app specific). As a whole, the 18 apps obtained a mean score for quality of 3.17 (SD 0.75). On average, the best-rated section was functionality (mean 3.92, SD 0.72), followed by esthetics (mean 3.29, SD 1.05) and engagement (mean 2.87, SD 1.14), whereas the worst-rated sections were app specific (mean 2.48, SD 1.00), information (mean 2.52, SD 0.82), and app subjective quality (mean 2.68, SD 1.22). The minimum score to be considered as a best-rated app was 3.73, according to the application of the tertiles previously described.

**Figure 3:**
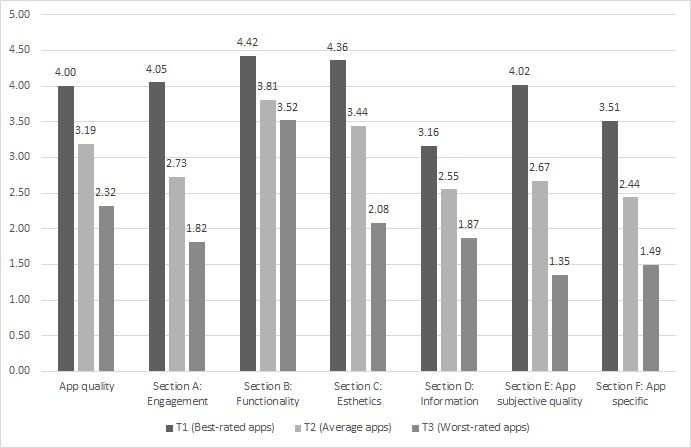
Differences in the mean MARS (Mobile App Rating Scale) scores between tertiles.

Figure 3 illustrates the differences found between tertiles regarding the scores of each section of the MARS. No major functionality (section B) differences between the best-rated and the worst-rated apps were found (*P*=.06), with a score range of 0.9 points. The highest differences were related to app subjective quality (section E), with a score range of 2.67 points (*P*<.001). Moreover, sections A (engagement), C (esthetics), and F (app specific) also had a score range greater than 2 points. All the differences were statistically significant except for section B.

**Table 2:** Description of the pain-related apps included in the study.

App name	Platform	Price (€)	Downloads	Developer	Affiliations
Manage My Pain (Lite & Pro)	Android	Free (Lite); €3.99 (Pro)	50,000-100,000	ManagingLife	Commercial
Diario de Dolor CatchMyPain (Lite & Pro)	Android-iOS	Free (Lite); €3.59 (Pro)	50,000-100,000	Sanovation AG	University
Mi registro de dolor	Android-iOS	Free	1000-5000	Subinprara Infotech Inc	Commercial
Pain Companion	Android	€1.09-€33.20 per element	5000-10,000	Sanovation AG	Commercial
OurHurt-Dolor Crónico	Android	Free	1000-5000	Labs Health Company	Commercial
My Pain Diary	Android	€3.66	1000-5000	DemoLab, LLC	Commercial
ACPA Pain Logs	Android-iOS	Free	500-1,000	ACPA	Commercial
Chronic Pain Diary	Android	Free	5000-10,000	Jet5	Commercial
Pain Tracker HD	Android-iOS	€0.89 per element	100-500	AppYourWay	Commercial
Painometer v2	Android	Free	1000-5000	Algos-Research on Pain	University
My Pain Diary & Symptom Tracker: Gold Edition	iOS	€5.49	1000-5000	Damon Lynn	Commercial
PainTrakr	iOS	Free	1000-5000	Black Slate Software Inc	Commercial
Pain Tracker & Diary by Nanulume	Android-iOS	€2.99	1000-5000	Nanolume, LLC	Commercial
GP Pain Help	Android	Free	1000-5000	Australian College of Rural & Remote Medicine	University
Pain Log	Android	Free	1000-5000	Raúl R	Commercial
Pain Score	Android	Free	500-1000	Trinstor	Commercial
Pain Rating Scales	Android	Free	1000-5000	ETZ	Commercial
Pain Treatment	Android	Free	10,000-50,000	Entertain2Dunia	Commercial

**Table 3:** Characteristics of the included pain-related apps.

App name	Focus (what the app targets)	Theoretical background/strategies	Technical aspects of app
Manage My Pain (Lite & Pro)	Physical health	Monitoring/tracking	Allows sharing (eg, Facebook, Twitter); allows password protection; requires log-in; sends reminders; needs Web access to function
Diario de Dolor. CatchMyPain (Lite & Pro)	Increase happiness/well-being emotions; reduce negative; anxiety/stress; physical health	Assessment; feedback; monitoring/tracking	Allows sharing (eg, Facebook, Twitter); allows password protection; requires log-in; sends reminders; needs Web access to function
Mi registro de dolor	Physical health	Assessment; feedback; monitoring/tracking	Allows password protection; requires log-in; sends reminders
Pain Companion	Increase happiness/well-being; goal setting; entertainment; relationships; physical health	Assessment; feedback; information/education; monitoring/tracking; advice tips/strategies/skills training; gratitude	Allows sharing (eg, Facebook, Twitter); has an app community; allows password protection; requires log-in; sends reminders; needs Web access to function
OurHurt-Dolor Crónico	Physical health	Monitoring/tracking; strengths	Allows sharing (eg, Facebook, Twitter); allows password protection; requires log-in
My Pain Diary	Depression; anxiety/stress; physical health	Monitoring/tracking	Allows sharing (eg, Facebook, Twitter)
ACPA Pain Logs	Physical health	Assessment; feedback; monitoring/tracking	Allows sharing (eg, Facebook, Twitter); allows password protection; requires log-in
Chronic Pain Diary	Physical health	Monitoring/tracking	—^a^
Pain Tracker HD	Physical health	Monitoring/tracking	Allows password protection; requires log-in; sends reminders
Painometer v2	Physical health	Assessment; monitoring/tracking	Allows sharing (eg, Facebook, Twitter)
My Pain Diary & Symptom Tracker: Gold Edition	Depression; anxiety/stress; physical health	Feedback; monitoring/tracking	Allows sharing (eg, Facebook, Twitter); has an app community
PainTrakr	Physical health	Monitoring/tracking	Sends reminders
Pain Tracker & Diary by Nanulume	Physical health	Monitoring/tracking	Requires log-in; sends reminders
GP Pain Help	Physical health	Assessment; information/education	—
Pain Log	Physical health	Monitoring/tracking	—
Pain Score	Physical health	Assessment; information/education	—
Pain Rating Scales	Physical health	Assessment; information/education; monitoring/tracking	—
Pain Treatment	Physical health	Assessment; information/education; advice/tips/strategies /skills training	Allows sharing (eg, Facebook, Twitter)

^a^The app does not have any of the technical aspects considered in the MARS.

**Table 4:** Mobile App Rating Scale (MARS) scoring of the pain-related apps.

App name	App quality, mean (SD)	T^a^	Section^b^, mean (SD)						User’s stars score
			A	B	C	D	E	F	
Pain Companion	4.35 (0.49)	T1	4.60 (0.55)	4.25 (0.05)	4.83 (0.06)	3.72 (1.62)	4.25 (0.50)	4.42 (0.52)	4.30
Manage My Pain (Lite & Pro)	4.22 (0.18)	T1	4.40 (0.89)	4.13 (0.03)	4.33 (0.03)	4.00 (0.38)	3.75 (1.00)	3.67 (0.82)	4.00
My Pain Diary & Symptom Tracker: Gold Edition	4.02 (1.08)	T1	4.30 (0.84)	4.50 (0.50)	4.83 (0.58)	2.43 (2.15)	4.38 (0.82)	3.42 (0.82)	N/A^c^
OurHurt-Dolor Crónico	3.88 (0.65)	T1	3.40 (1.14)	4.75 (0.05)	4.00 (0.58)	3.36 (1.50)	3.88 (0.50)	3.17 (0.52)	3.90
Pain Tracker & Diary by Nanulume	3.79 (0.43)	T1	3.50 (1.52)	4.38 (0.50)	3.83 (0.58)	3.43 (1.70)	3.88 (0.82)	3.33 (0.63)	2.50
My Pain Diary	3.73 (1.17)	T1	4.10 (0.71)	4.50 (0.50)	4.33 (1.00)	2.00 (1.51)	4.00 (0.82)	3.08 (0.55)	4.20
Mi registro de dolor	3.60 (1.20)	T2	4.30 (0.84)	3.75 (0.50)	4.50 (0.58)	1.86 (1.57)	3.50 (1.50)	3.34 (1.26)	3.70
Diario de Dolor. CatchMyPain (Lite & Pro)	3.35 (0.48)	T2	3.60 (0.45)	3.50 (0.58)	3.67 (0.07)	2.64 (1.98)	2.88 (0.50)	2.75 (0.98)	4.00
ACPA Pain Logs	3.16 (0.41)	T2	2.40 (0.55)	3.00 (0.50)	3.33 (0.06)	2.64 (1.07)	3.75 (0.50)	2.25 (0.41)	3.00
GP Pain Help	3.10 (0.90)	T2	2.30 (1.34)	4.38 (0.50)	3.00 (0.58)	2.71 (2.37)	2.13 (0.96)	2.83 (1.22)	4.80
Pain Log	3.08 (1.03)	T2	1.70 (1.30)	4.13 (0.04)	3.50 (1.00)	3.00 (1.50)	2.00 (1.26)	1.83 (0.52)	4.40
Pain Rating Scales	2.83 (0.89)	T2	2.10 (0.02)	4.13 (0.82)	2.66 (0.03)	2.43 (1.11)	1.75 (0.05)	1.67 (0.55)	4.30
Painometer v2	2.65 (0.53)	T3	2.10 (1.30)	3.25 (0.58)	2.33 (0.58)	2.93 (0.69)	1.75 (0.82)	1.00 (0.00)	4.10
Chronic Pain Diary	2.61 (0.58)	T3	2.40 (0.55)	3.38 (0.03)	2.00 (0.00)	2.64 (1.50)	1.50 (0.58)	1.83 (0.41)	3.20
Pain Score	2.41 (1.59)	T3	1.60 (0.89)	4.75 (1.00)	2.00 (0.00)	1.29 (1.50)	1.00 (0.00)	1.00 (0.00)	3.50
Pain Treatment	2.36 (1.35)	T3	1.20 (0.02)	4.25 (0.50)	2.33 (0.58)	1.64 (1.21)	1.38 (0.50)	2.33 (0.82)	3.00
PainTrakr	2.17 (1.04)	T3	1.90 (0.55)	3.63 (0.50)	2.00 (0.58)	1.14 (0.95)	1.38 (0.04)	1.33 (0.03)	N/A
Pain Tracker HD	1.74 (0.14)	T3	1.70 (0.55)	1.88 (0.50)	1.83 (0.58)	1.57 (1.29)	1.13 (0.50)	1.42 (0.75)	1.00

^a^T: tertile. Tertile legend: T1: best-rated apps; T2: average apps; T3: worst-rated apps.

^b^A: Engagement; B: Functionality; C: Esthetics; D: Information; E: App subjective quality; F: App specific.

^c^N/A: not applicable.

## Discussion

### Principal Results

This paper presents a systematic search and evaluation of apps related to pain in the App Store and Play Store. First, it is important to note that the mobile app market is very volatile, unpredictable, and constantly changing, and it is likely that the situation at the time of publication of this paper is not exactly the same as the one presented here. Indeed, during the completion of this study, we detected some changes in the market. Specifically, we had to remove an app from our study because it no longer worked (or even existed in the store) when we tried to use it again to solve some doubts over its rating. Despite this, we present here what, as far as we are concerned, are the most accurate results regarding the quality of the pain apps available at present because it includes an assessment using a validated tool such as the MARS.

Before discussing the scores obtained and the quality of the apps in general, there are some aspects that we would first like to highlight due to their importance in the scientific field: the theoretical support of the apps and the need for randomized controlled trials to test them on people. There is one specific item in the MARS assessing the “evidence base,” which explores the extent to which the app has been scientifically tested. Although the results of the MARS are shown in terms of dimensions and not specific items, it is important to mention here that only two of the apps (Manage My Pain Lite & Pro and Painometer v2) had been tested or trialed to some extent, showing positive or partially positive results. Surprisingly, the latter is not very well positioned in terms of its score on the MARS (it belongs in the worst-rated apps tertile), for reasons that will be discussed later. However, above other criteria, it is crucial for a health app to be tested if it aspires to become a useful tool for health professionals and patients, ensuring safety and good functioning. This is particularly important when dealing with new technology, as discussed in the Introduction [19,20]. In this regard, many studies that propose the use of new technologies conclude that more evidence is needed to support their use [23-26]. A possible explanation for the lack of scientific support could be that most of the apps are commercial (in our sample, only three came from academia), which suggests the need to promote the development of apps from scientific institutions.

Another important issue to bear in mind is security and privacy. A recent article by Papageorgiou et al [27] highlights the sensitive nature of the data collected by health-related apps and the need to follow standards of good practice and comply with data protection laws. Although not restricted to pain apps, their conclusions are alarming because most health apps do not even comply with the law. Regarding our results, MARS collects information on security and privacy on two occasions: it asks if the app allows password protection and if it requires log-in. None of them is part of the scored sections, and they do not cover security after the information is collected by the app. Half of the apps included in this evaluation allowed password protection and/or required log-in. However, we cannot know to what extent data protection laws and standards of good practice are met, although the answers to these two MARS items, as well as the conclusions of Papageorgiou et al, do not give great cause for optimism in this regard. Future apps should consider this aspect.

An ideal app, apart from being based on scientific evidence and respecting the law and privacy, should be user friendly, attractive, simple, and functional, and exploit the sensors and other capabilities of the devices for the benefit of the patient. However, we already argued in the Introduction that the use of certain technologies or apps could sometimes be harmful, although a recent review by Lee et al [28] states that mobile health (mHealth) intervention studies show promising aspects such as improving self-management and some health indicators. In this sense, it would be desirable to have the authorization of the corresponding health authority to recommend (and even finance) the use of certain devices and/or apps. In countries such as the United Kingdom, there is a policy of promoting and sometimes financing wearable mHealth devices for chronic pain management [29], although the situation is not the same in other countries, where this goal seems distant.

Regarding the quality of the apps included in this study, we can say that they are mostly good, although the best scores correspond to technical aspects of the app itself, such as “functionality,” while the worst relate more to what is offered to the user and their opinion (“information” and “app subjective quality”). This means that the apps seem to be more or less well designed but fail to fully convince the users. Surprisingly (and apparently in contrast to the previous statement), we observed in our sample that 68.75% of the users’ ratings (via the stars system) in the corresponding stores were higher than those obtained with the MARS. However, it is a single and completely subjective score based on the criteria of the users themselves compared to the result of having applied a validated assessment tool whose results are more reliable. In any case, our results show that it would be necessary to improve less valued aspects such as the information offered to the user, which turns out to be a crucial aspect in this kind of app.

It is important to note that some apps are very specific or perform a single task and this could lead to a decrease in their score in the MARS. It is necessary to bear this in mind when making a critical reading of the classification of the apps that we present here. Some of them might be worse rated not because they were actually worse, but because a high score could not be given to some items of the MARS; these apps can still be excellent in other aspects. For this reason, it is important to observe not only the global score (used to determine the tertiles), but the scores in each dimension. In fact, in our sample we found apps that are among the worst rated but have better scores in dimensions such as “functionality” than the best-rated apps. This study does not intend to make recommendations about what app to use, but merely to show reliable information that can be used by the reader according to their own criteria and all the aspects discussed.

In view of this, one might wonder what determines how an app achieves a higher global score. That is, which aspects characterize a highly valued app or make an app more highly valued than another? In a way, the comparison carried out between tertiles can give us some clues about this because it highlights the differences between the three groups. The main differences were found in app subjective quality, engagement, and esthetics, so these should be the aspects to improve in order to “climb positions” in the app ranking. Nevertheless, in this case, as they are apps intended for health care, these aspects must be secondary, always less important than other relevant aspects already mentioned, such as the scientific basis, security, and privacy.

### Limitations

Finally, this study has the usual limitations of these types of studies, and particularly those due to the nature of the items studied (mobile apps). We highlight the possibility of having missed some pain apps that did not contain the word “pain” in its title or its description. Another possible limitation is that the reliability of the MARS was originally piloted on iPhone apps. However, the same authors state that the scale has been applied to multiple Android apps, finding no compatibility issues. Also, the apps market is constantly changing, and this fact can significantly shorten the validity period of this evaluation. This, in turn, can also be seen as a strength, as this is the most recent update at the time of its publication, and hence the closest approximation to the current situation of pain-related apps. Moreover, unlike other authors, we use an adequate tool to measure the quality of the apps, which is a substantial improvement over previous reviews. Additionally, we include paid apps, which are likely to have different characteristics, options, and ratings, and which are not always included in other reviews, possibly leading to bias.

### Conclusions

The pain-related apps that are currently available in the market are of a certain quality, mainly regarding their technical aspects, although they fail to offer information and have an impact on the user. On the other hand, the vast majority of apps are not based on scientific evidence, have not been rigorously tested, and the confidentiality of the information collected is not guaranteed. Future apps would need to improve these aspects, exploit the capabilities of the latest devices, and comply with some other requirements, such as being user friendly, attractive, simple, and functional for the benefit of the patient. These conclusions provide, in our opinion, a more objective perspective than the previous reviews in which no validated tools were used to measure the quality.

## Abbreviations

ANOVA: analysis of variance
IASP: International Association for the Study of Pain
MARS: Mobile App Rating Scale
mHealth: mobile health

## References

[1] Williams AC, Craig KD (2016). Updating the definition of pain. Pain.

[2] Merskey H, Bogduk N, Merskey H, Bogduk N (1994). A current list definitions and notes on usage. Part III: pain terms. Classification of chronic pain. Descriptions of chronic pain syndromes and definitions of pain terms.

[3] Dueñas M, Salazar A, Ojeda B, Fernández-Palacín F, Micó JA, Torres LM, Failde I (2015). A nationwide study of chronic pain prevalence in the general spanish population: identifying clinical subgroups through cluster analysis. Pain Med.

[4] Dueñas M, Ojeda B, Salazar A, Failde I, Atiq M (2010). Health related quality of life in coronary patients. Recent Advances in Cardiovascular Risk Factors.

[5] Dueñas M, Ojeda B, Salazar A, Mico JA, Failde I (2016). A review of chronic pain impact on patients, their social environment and the health care system. J Pain Res.

[6] Azevedo LF, Costa-Pereira A, Mendonça L, Dias CC, Castro-Lopes JM (2012). Epidemiology of chronic pain: a population-based nationwide study on its prevalence, characteristics and associated disability in Portugal. J Pain.

[7] Breivik H, Collett B, Ventafridda V, Cohen R, Gallacher D (2006). Survey of chronic pain in Europe: prevalence, impact on daily life, and treatment. Eur J Pain.

[8] Leadley RM, Armstrong N, Lee YC, Allen A, Kleijnen J (2012). Chronic diseases in the European Union: the prevalence and health cost implications of chronic pain. J Pain Palliat Care Pharmacother.

[9] Grol-Prokopczyk H (2017). Sociodemographic disparities in chronic pain, based on 12-year longitudinal data. Pain.

[10] Birke H, Kurita GP, Sjøgren P, Højsted J, Simonsen MK, Juel K, Ekholm O (2016). Chronic non-cancer pain and the epidemic prescription of opioids in the Danish population: trends from 2000 to 2013. Acta Anaesthesiol Scand.

[11] Gatchel RJ, Peng YB, Peters ML, Fuchs PN, Turk DC (2007). The biopsychosocial approach to chronic pain: scientific advances and future directions. Psychol Bull.

[12] de Sola H, Salazar A, Dueñas M, Ojeda B, Failde I (2016). Nationwide cross-sectional study of the impact of chronic pain on an individual's employment: relationship with the family and the social support. BMJ Open.

[13] Ojeda B, Dueñas M, Salazar A, Mico JA, Torres LM, Failde I (2018). Factors influencing cognitive impairment in neuropathic and musculoskeletal pain and fibromyalgia. Pain Med.

[14] Gatchel RJ (2004). Comorbidity of chronic pain and mental health disorders: the biopsychosocial perspective. Am Psychol.

[15] Ojeda B, Salazar A, Dueñas M, Torres LM, Mico JA, Failde I (2014). The impact of chronic pain: the perspective of patients, relatives, and caregivers. Fam Syst Health.

[16] Grundy Q, Held F, Bero L (2017). Tracing the potential flow of consumer data: a network analysis of prominent health and fitness apps. J Med Internet Res.

[17] Stoyanov SR, Hides L, Kavanagh DJ, Zelenko O, Tjondronegoro D, Mani M (2015). Mobile app rating scale: a new tool for assessing the quality of health mobile apps. JMIR Mhealth Uhealth.

[18] Portelli P, Eldred C (2016). A quality review of smartphone applications for the management of pain. Br J Pain.

[19] Rosser BA, Eccleston C (2011). Smartphone applications for pain management. J Telemed Telecare.

[20] Buijink AW, Visser BJ, Marshall L (2013). Medical apps for smartphones: lack of evidence undermines quality and safety. Evid Based Med.

[21] Morera E, de la Torre I, Garcia-Zapirain B, Lopez-Coronado M, Arambarri J (2016). Security recommendations for mHealth apps: elaboration of a developer's guide. J Med Syst.

[22] Kitagawa M, Gupta A, Cozza R, Glenn D, Maita K, Durand I, Lakehal B, Escherich M, Tay L, Tsai T, He E, Nguyen T, Zimmermann A, Jump A, Sato A, Atwal R, Lu C (2017). Market share: final PCs, ultramobiles and mobile phones, all countries, 4Q16 update. Gartner Technical Report.

[23] Jonassaint C, Rao N, Sciuto A, Switzer G, De Castro L, Kato G, Jonassaint H, Hammal Z, Shah N, Wasan A (2018). Using abstract animations as an innovative technology-based approach to measuring pain in adults. J Med Internet Res.

[24] Lalloo C, Shah U, Birnie KA, Davies-Chalmers C, Rivera J, Stinson J, Campbell F (2017). Commercially available smartphone apps to support postoperative pain self-management: scoping review. JMIR Mhealth Uhealth.

[25] Wu Y, Yao X, Vespasiani G, Nicolucci A, Dong Y, Kwong J, Li L, Sun X, Tian H, Li S (2017). Mobile app-based interventions to support diabetes self-management: a systematic review of randomized controlled trials to identify functions associated with glycemic efficacy. JMIR Mhealth Uhealth.

[26] Covolo L, Ceretti E, Moneda M, Castaldi S, Gelatti U (2017). Does evidence support the use of mobile phone apps as a driver for promoting healthy lifestyles from a public health perspective? A systematic review of Randomized Control Trials. Patient Educ Couns.

[27] Papageorgiou A, Strigkos M, Politou E, Alepis E, Solanas A, Patsakis C (2018). Security and privacy analysis of mobile health applications: the alarming state of practice. IEEE Access.

[28] Lee J, Choi M, Lee SA, Jiang N (2018). Effective behavioral intervention strategies using mobile health applications for chronic disease management: a systematic review. BMC Med Inform Decis Mak.

[29] Wicklund E (2018). mHealth Intelligence.

